# 

**Published:** 1911-02

**Authors:** 


### BOOKS RECEIVED

A Manual of Pharmacy. By M.F. DeLorme, M.D., Ph.G., Lecturer on Pharmacy and Pharmacology in Long Island College Hospital, New York. Second edition. 12 mo, pp. 207. With 19 illlustrations. Philadelphia: P. Blakiston's Son's & Co. 1911. (Cloth, $1.25 net.)

Treatise on Diagnostic Methods. By Professor Dr. Herman Sahli, Director of the Medical Clinic, University of Bern. Edited by Nathaniel Bowditch Potter, M.D., Assistant Professor of Clinical Medicine at Columbia University, New York. Second edition. Octavo, pp. 1229. Ilustrated. Philadelphia and London: W. B. Saunders Co. 1911. (Cloth, $6.50.)

Vaginal Celiotomy. By S. Wyllis Bandler, M.D., Fellow of the American Association of Obstetricians and Gynecologists; Adjunct Professor of Diseases of Women, New York Post-Graduate Medical School and Hospital. Octavo, pp. 450, with 148 original illustrations. Philadelphia and London: W. B. Saunders Co. 1911. (Cloth, $5.00.)

Anatomic Histological Processes of Bright's Disease and their Relation to the Functional Changes. Lectures delivered in the Russell Sage Institute of Pathology, City Hospital, New York, during the winter of 1909. By Horst Oertel, Director. Octavo, pp. 239. Illustrated. Philadelphia and London: W. B. Saunders Co. 1910. (Cloth, $5.00.)

Induced Cell-Reproduction and Cancer. By Hugh Campbell Ross, M.R.C.S. (Eng.), L.R.C.P. (Lond.), Director of Special Researches at Royal Southern Hospital, Liverpool. Octavo, pp. 453. Illustrated. Philadelphia: P. Blakiston's Son & Co. 1911. (Cloth, $4.50.)

Bismuth Paste in Chronic Suppurations. By Emil G. Beck, M.D., Surgeon to the North Chicago Hospital. With an Introduction by Carl Beck, M.D. Octavo, pp. 237. Illustrated. St. Louis: C. V. Mosby Co. 1910. (Cloth, $2.50.)

Principles of Public Health. By Thomas D. Tuttle, M.D. Secretary of the State Board of Health of Montana. Yonkers: World Book Co. (Cloth, 50 cents.)

Primer of Hygiene. By John W. Ritchie, Professor of Biology, College of William and Mary, Va., and Joseph S. Caldwell, Tenn. Yonkers: World Book Co. (Cloth, 40 cents.)

Principles of Therapeutics. By A. Manquat, National Correspondent to the Academy of Medicine. Translated by M. Simbad Gabriel, M.D. Octavo, pp. 298. New York and London: D. Appleton & Co. 1910. (Cloth, $3.00.)

Report of the Commissioner of Education. For the year ended June 30, 1910. Volume 1. Washington: Government Printing Office.

Publications of the Massachusetts General Hospital. Vol. II. No. 2.

The Care and Training of Children. By LeGrand Kerr, M.D., Brooklyn. 12 mo. New York: Funk & Wagnalls Company. (Cloth, 75 cents net; by mail, 82c.)

The Practical Medicine Series. Ten volumes. Under the general editorial charge of Gustavus P. Head, M. D. Vol. IX. Skin and Venereal Diseases. Edited by W. C. Baum, M.D., and Harold N. Moyer, M.D. Series 1910. Chicago: The Year Book Publishers. (Price, $1.25; entire series, $10.00.)

